# Complementing the topsoil information of the Land Use/Land Cover Area Frame Survey (LUCAS) with modelled N_2_O emissions

**DOI:** 10.1371/journal.pone.0176111

**Published:** 2017-04-27

**Authors:** Emanuele Lugato, Lily Paniagua, Arwyn Jones, Wim de Vries, Adrian Leip

**Affiliations:** 1 European Commission, Joint Research Centre (JRC), Sustainable Resources Directorate, Ispra, Varese, Italy; 2 Wageningen University and Research, Environmental Systems Analysis Group, Wageningen, The Netherlands; 3 Wageningen University and Research, Environmental Research (Alterra), Wageningen, The Netherlands; Kerala Forest Research Institute, INDIA

## Abstract

Two objectives of the Common Agricultural Policy post-2013 (CAP, 2014–2020) in the European Union (EU) are the sustainable management of natural resources and climate smart agriculture. To understand the CAP impact on these priorities, the Land Use/Cover statistical Area frame Survey (LUCAS) employs direct field observations and soil sub-sampling across the EU. While a huge amount of information can be retrieved from LUCAS points for monitoring the environmental status of agroecosystems and assessing soil carbon sequestration, a fundamental aspect relating to climate change action is missing, namely nitrous oxide (N_2_O) soil emissions. To fill this gap, we ran the DayCent biogeochemistry model for more than 11’000 LUCAS sampling points under agricultural use, assessing also the model uncertainty. The results showed that current annual N_2_O emissions followed a skewed distribution with a mean and median values of 2.27 and 1.71 kg N ha^-1^ yr^-1^, respectively. Using a Random Forest regression for upscaling the modelled results to the EU level, we estimated direct soil emissions of N_2_O in the range of 171–195 Tg yr^-1^ of CO_2eq_. Moreover, the direct regional upscaling using modelled N_2_O emissions in LUCAS points was on average 0.95 Mg yr^-1^ of CO_2eq_. per hectare, which was within the range of the meta-model upscaling (0.92–1.05 Mg ha^-1^ yr^-1^ of CO_2eq_). We concluded that, if information on management practices would be made available and model bias further reduced by N_2_O flux measurement at representative LUCAS points, the combination of the land use/soil survey with a well calibrated biogeochemistry model may become a reference tool to support agricultural, environmental and climate policies.

## Introduction

Following decisions 1445/2000/EC and 2066/2003/EC [1,2], the statistical office of the European Union (Eurostat) has established a regular survey to monitor changes in land use over time across the European Union (EU). This survey, known as Land Use and Coverage Area frame Survey (LUCAS), classified the land use and cover for around 1’000’000 geo-referenced locations by remote sensing, of which around 25% were control points visited by field surveyors (currently, this equates to 270’000 sites) [1]. In 2009, the scope of the survey was extended by including a topsoil component for 10% of the control points (c. 20’000 samples) that aimed to create a harmonised and comparable dataset of physical and chemical properties of topsoil across the EU [3]. In order to monitor the impact of land related policies on soil conditions and to support new policy development, the soil survey was repeated in 2015 and is planned to be undertaken about every 3–6 years. This data collection exercise is fundamental to assess the impact of implemented policies on soil quality, mainly in view of changes in pools of carbon, nitrogen, phosphorous and base cations, in addition to its function to act as sink for atmospheric carbon dioxide. However, the data do not cover the emission of nitrous oxide (N_2_O) which is a highly policy-relevant biogeochemical nitrogen flow in view of climate action (climate smart agriculture), as it may (partly) offset the effect of soil carbon sequestration actions [4].

Nitrogen (N) is needed in agricultural systems to produce food, fuel and fiber and, despite being a major constituent of the atmosphere as dinitrogen, it is only available to plants and other organisms once it is ‘activated’ [5]. Once activated, reactive N cascades through the environment potentially contributing to a number of environmental issues, including air pollution, soil acidification, fresh and coastal water eutrophication, and global warming [6–8]. Anthropogenic fixation of N through the Haber—Bosch process is today the largest source of new reactive N, estimated to be near 120 Tg N yr^-1^ in 2010 [9], of which about 85% is used as agricultural fertilizer (105 Tg N yr^-1^), with the remaining part used as feedstock for industrial processes (http://www.fertilizerseurope.com/). Use of synthetic N fertilizer in Europe was estimated at 10% of the global use (10.5 Tg N yr^-1^) in 2010; however, at a continental scale, the import of N with feed to Europe is at 2.7 Tg N yr^-1^, representing another significant source of reactive N [10]. Amongst the consequences of the N-cascade are the emissions of N_2_O, the third most important long-lived greenhouse gas, which contributes 5% to global anthropogenic radiative forcing [11].

Terrestrial sources of N_2_O are dominated by microbial production in soils, but the underlying processes are highly complex and depend on the interaction between soil characteristics, weather, land use and management practices [12]. As a consequence, N_2_O fluxes from soils are characterized by an inherently high spatial and temporal variability that makes extrapolation to annual fluxes at regional or national scale difficult [13,14]. At the same time, field measurements of N_2_O fluxes are laborious and expensive to carry out, which means that the number of suitable data sets to derive N_2_O emission factors is usually insufficient [15]. Accordingly, N_2_O emission estimates are amongst the largest source of uncertainty in national greenhouse gas inventories [16].

Process-based models, with different degrees of complexity, can be used to estimate N_2_O emissions from soil in varying ecosystems and, thus, reduce uncertainty around the estimates. Complex models represent the production, consumption and diffusion of N during the nitrification and denitrification processes. The most widely used process-based models to quantify agricultural GHG emissions for landscapes or regions are DNDC, EPIC/APEX and DayCent, each of which are regarded as being well validated [17]. However, due to the model complexity, the volume of input data, the high level of user expertise and computation resources required, the use of these models for large-scale assessments is limited. Even though these model have been applied on a national and even continental scale [18], there is a trade-off between model complexity and data availability, thus making the results at small geographical scales highly uncertain. This is a clear drawback since the use of default (IPCC tier 1) or soil and land use dependent emission factors (IPCC tier 2) do not capture in sufficient detail the impacts of land use, climate and soil on N_2_O emissions nor demonstrate the potential for reducing N_2_O emissions. It is therefore important to develop a methodology that helps to bridge the generic approach using default or specific IPCC emission factors (tier 1 and 2), and the more sophisticated approach using complex process oriented biogeochemical models (tier 3). Simplified approaches often consist of running a model on representative sites, then constructing a meta-model (a second level of abstraction where the input-output relationship is simplified on the basis of a significant statistical relationship) to upscale the results [19–22]. This allows scenario analysis and decision support systems to be easily developed at regional levels, since it requires fewer parameters and shorter computational times than the original model. Despite the relevance of using meta-models, use of the original model represents an advantage as soon as appropriate input data are available, being a prerequisite for reliable model results.

Within this context, the aim of this research was to demonstrate a framework that can be used to improve estimates of N_2_O emissions from agricultural soils at continental scales using the original DayCent model with adequate input data. We used one of largest land-use/soil inventory framework in the EU to parameterize the DayCent model with the aim to: 1) provide N_2_O soil flux estimates at LUCAS locations, including an assessment of their sensitivity to the most important parameters; 2) create a meta-model with DayCent inputs and outputs to spatialize the results at regional level and 3) compare regional estimates of N_2_O emissions derived directly from LUCAS point simulations with more complex upscaling methodologies. The final objective is to develop a proposal on how the LUCAS survey could be used to improve agricultural N_2_O emission estimates.

## Materials and methods

**Fig 1:**
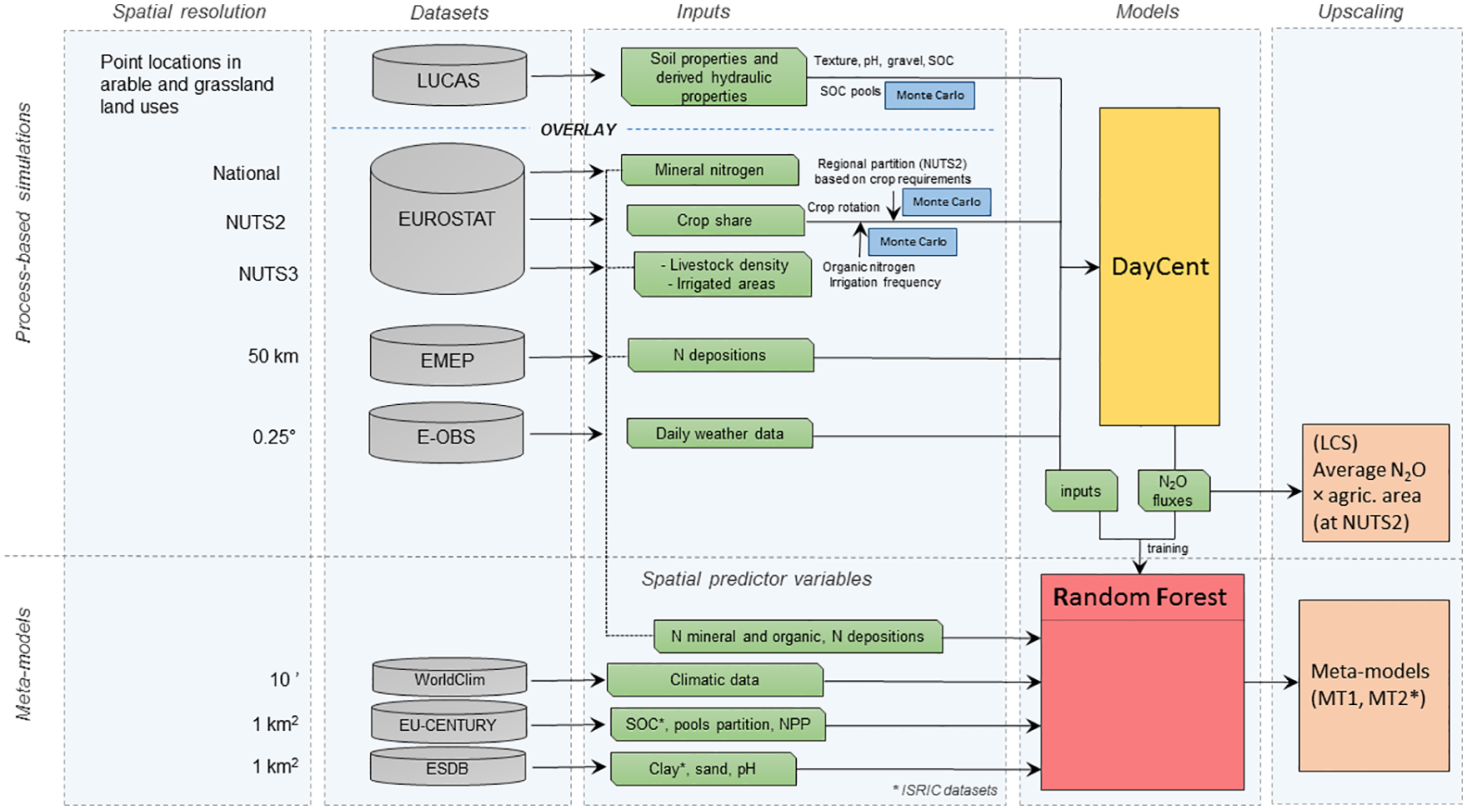
Flow chart showing the datasets utilized and their spatial resolution, the inputs derived, the model integration and the upscaling process.

The dataset characteristics and inputs derived, the models integration and the upscaling processes are summarized in the flow chart Fig 1. The first step was to run the DayCent biogeochemistry model on the LUCAS points that were classified as either arable or grassland, using direct information from the survey and supported by other EU datasets (Fig 1).

In the second phase, we developed a meta-model from inputs and model outputs based on the Random Forest regression approach, which was upscaled by a set of spatial predictor variables, including high resolution spatial layers from published studies. More details of the methodology are provided in the following paragraphs.

### LUCAS soil dataset

Through a combination of remote sensing and direct field observations, the LUCAS survey gathers harmonized data on land use and cover across the EU, together with changes over time. It includes a soil component based on 10% of the survey’s control points, providing in 2009 approximately 20’000 sampling locations. Topsoil samples (0–20 cm) were taken from all land use and land cover types, with a slight bias for agricultural areas [3]. The samples were analysed in a single ISO-certified laboratory for: percentage of coarse fragments, particle size distribution (% clay, silt and sand content), pH (in CaCl_2_ and H_2_O), organic carbon content (g kg^-1^), carbonate content g kg^-1^), phosphorus content (mg kg^-1^), total nitrogen content (g kg^-1^), extractable potassium content (mg kg^-1^) and cation exchange capacity (cmol(+) kg^-1^). Further information and data accessibility can be found at: http://esdac.jrc.ec.europa.eu/projects/lucas.

For the purpose of this study we included only the agricultural land uses classified as arable/ rotational forage crops (codes B11 to B55 of the LUCAS land cover classification scheme—LC1) and the grassland categories (E10 and E20).

### DayCent model

DayCent, the daily time-step version of the Century biogeochemical model [23], was designed to simulate soil C dynamics, nutrient flows (N, P, S), and trace gas fluxes (CO_2_, CH_4_, N_2_O, NO_x_, N_2_) between soil, plants and the atmosphere [24]. Sub-models include soil water content and temperature by layer, plant production and allocation of net primary production (NPP), decomposition of litter and soil organic matter, mineralization of nutrients, N gas emissions from nitrification and denitrification, and CH_4_ oxidation in non-saturated soils. The model is driven by daily meteorological data, site characteristics and the main agricultural management practices such as crop rotation, tillage, grazing, irrigation, organic and mineral fertilisation inputs.

### Model inputs and model application

The inputs needed to run the DayCent model were derived by using: 1) information on soil properties available for LUCAS points (type I), which was considered very accurate and directly used as input parameters without an uncertainty range; 2) information from official statistics not available at point-level (type II) and subject to uncertainty analysis, depending on the sensitivity of modelled N_2_O emissions to their variation.

Type I information included the initial soil organic carbon content (SOC), particle size distribution and pH. Hydraulic properties such as field capacity, wilting points and saturated hydraulic conductivity were estimated using a pedotransfer rule [25], based on texture and SOC content. Hydraulic properties were corrected for the presence of stones according to the formula [1-(Rv/100)], where Rv is the rock fragment content by volume. Soil bulk density was also calculated with an empirically-derived pedotransfer function [26].

Type II information was derived from official statistics (Eurostat, http://ec.europa.eu/eurostat/web/main/home) and included crop shares at NUTS2 level (administrative borders, which represent the EU basic regions for the application of regional policies), livestock density and irrigated areas at NUTS3 level, and mineral N consumption at national level (Fig 1). The data on crop shares, irrigated areas and livestock density were used to derive regional crop rotations, irrigation frequency and organic fertilizer (manure) inputs (see Fig 1). The methodology for obtaining those inputs has been described in a recent pan European SOC modelling study with the Century model [27] and the resulting maps from this study were used. The amount of mineral N at national level was partitioned according to the regional crop rotations and agronomical crop requirements (the fertilizer amounts applied at LUCAS points are reported in S1 and S2 Figs). Since the modelled N_2_O fluxes are sensitive to N availability, a probability density function (PDF) with a mean and variance equal to 1 and 0.2, respectively, was used to generate the uncertainty of N fertilization inputs. The model was run 50 times on each LUCAS point multiplying the derived inputs by the randomly sampled PDF values (i.e. a Monte Carlo approach).

As with other widely used biogeochemistry models (e.g. DNDC), the parameter most influencing N_2_O fluxes in DayCent is the SOC content [28,29]. To avoid poor model performance in organic soils, LUCAS locations with a SOC content over 20% were excluded. This removed 137 of the 11765 points in the LUCAS agricultural subset (about 1.1%).

The starting year of the simulation was set at 2009 (year of LUCAS sampling), so that initial SOC values corresponded to the measured ones. The initial passive:total SOC ratio was derived from a large-scale application of the Century model [27,30], which is highly consistent with the DayCent model structure and where a long-term spin-up was made, since the passive pool has a turnover time ranging from 400–2000 years and is not a measurable parameter. To estimate the impact of the uncertainty in SOC initialization on N_2_O emissions, we ran DayCent with a ‘passive pool’ distribution multiplying the passive:total SOC ratio with a randomly sampled PDF with a mean and standard deviation of 1 and 0.2, respectively.

Atmospheric N deposition data were obtained from average values (2006–2010) of the EMEP model (rv 4.5) [31] results, providing spatially distributed (50 km^2^ resolution) wet and dry deposition. Meteorological data were taken from the E-OBS gridded dataset (http://www.ecad.eu/download/ensembles/downloadversion11.0.php#datafiles). The dataset provided daily data of maximum and minimum temperature and precipitation on a grid of 0.25° resolution.

The model was run from 2009 to 2014 but the outputs were taken after 2010, allowing a one-year equilibrium of the fast N and SOC pools and water status in the soil profile. About 11’628 points distributed in two land uses (arable and grassland) were simulated, for a total of 581’400 runs considering the uncertainty analysis. A Linux server with 24 core and 64GB of RAM allowed a reasonable computational time of about 11 hours.

### Crops simulation and validation

For the arable land use, the following crops were available in the DayCent model: winter and spring barley, winter and spring wheat, forage and grain maize, oilseed rape, potato, sugar beet, soybean, sunflower, pulses and cotton. The planting and harvesting date for each crop was based on the crop calendar map, available at the SAGE Center (https://nelson.wisc.edu/sage/data-and-models/crop-calendar-dataset/index.php) [32].

The LUCAS survey does not provide information about the specific management, therefore conventional agro-techniques were assumed to be in place; these included a primary (mouldboard) and secondary tillage and mineral N application split in two events (depending on crop type).

Crops yields at NUTS2 level were collected from the Eurostat portal and used as comparison with modelled yields, the latter aggregated at the same NUTS2 level. Crop yields from Eurostat were converted first to dry matter, utilizing the moisture content indicated by the “Eurostat Handbook for Annual Crop Statistics” and, second, to carbon (multiplying by 0.45) to match the same modelled units. Consequently, some calibration was made on the ‘potential above ground production coefficient’ for maize, potato, and sugar beet in order to reduce the deviation with measured data. All other crop parameters, including those controlling SOC decomposition or N fluxes were default values as given in the DayCent library.

### Regional upscaling

Two methods were applied to extend the results from LUCAS points to the whole agricultural area at the level of administrative regions (NUTS2) (Fig 1):

1. multiplication of average N_2_O emission rates simulated at all LUCAS points in each region, with its total agricultural area derived from the Corine Land Cover;
2. application of a meta-model by a Random Forest regression, trained to predict N_2_O emissions with a subset of variables.

In the latter approach, firstly, the Random Forest was developed from simulation inputs and outcomes and, secondly, it was applied regionally using two different sets of spatial predictor variables to assess the uncertainty associated with input data.

The ‘RandomForest’ library of R-core (https://www.r-project.org/) was used to predict N_2_O annual fluxes (kg N ha^-1^ yr^-1^) from the following predictors: clay (%), sand (%), SOC stock (Mg C ha^-1^), PTR (passive to total SOC ratio), soil pH, N-org (annual organic N fertilization), N-min (annual mineral N fertilization), atmospheric N deposition, NPP (net primary production), MxAT (maximum annual temperature), MnAT (minimum annual temperature), rain (annual precipitation). To evaluate the meta-model performances, the dataset derived from DayCent outcomes was split into a training and testing data, randomly sampling 75% of the original records.

The upscaling of the fitted Random Forest model was done by: 1) a meta-model MT1, where most of the spatial predictors were taken from the pan European SOC modelling application, previously developed with Century model [27,30] and 2) a meta-model MT2, where the two most explaining variables (SOC stock and clay content, see Results) were substituted in MT1 by the corresponding high resolution soil maps from the ISRIC repository (http://www.isric.org/content/soilgrids) [33].

In upscaling the model results (see Fig 1), use was made of soil data as given in the European soil database (ESDB; http://esdac.jrc.ec.europa.eu/resource-type/european-soil-database-soil-properties) and climate data as given by WorldClim (http://www.worldclim.org/current).

The difference (Var) in meta-model predictions were evaluated against the direct aggregation of LUCAS point simulations as follow:

$${\text{Var}}_{\text{n}}{\text{= (MT}}_{\text{n}}{\text{- LCS}}_{\text{n}}{\text{)/LCS}}_{\text{n}}*100$$ (1)

were MT is the average N_2_O emission of all inferred pixels at NUTS2 level (n) and LCS is the average of LUCAS-DayCent simulated values in the respective NUTS2 regions.

## Results

### Crop yield

**Fig 2:**
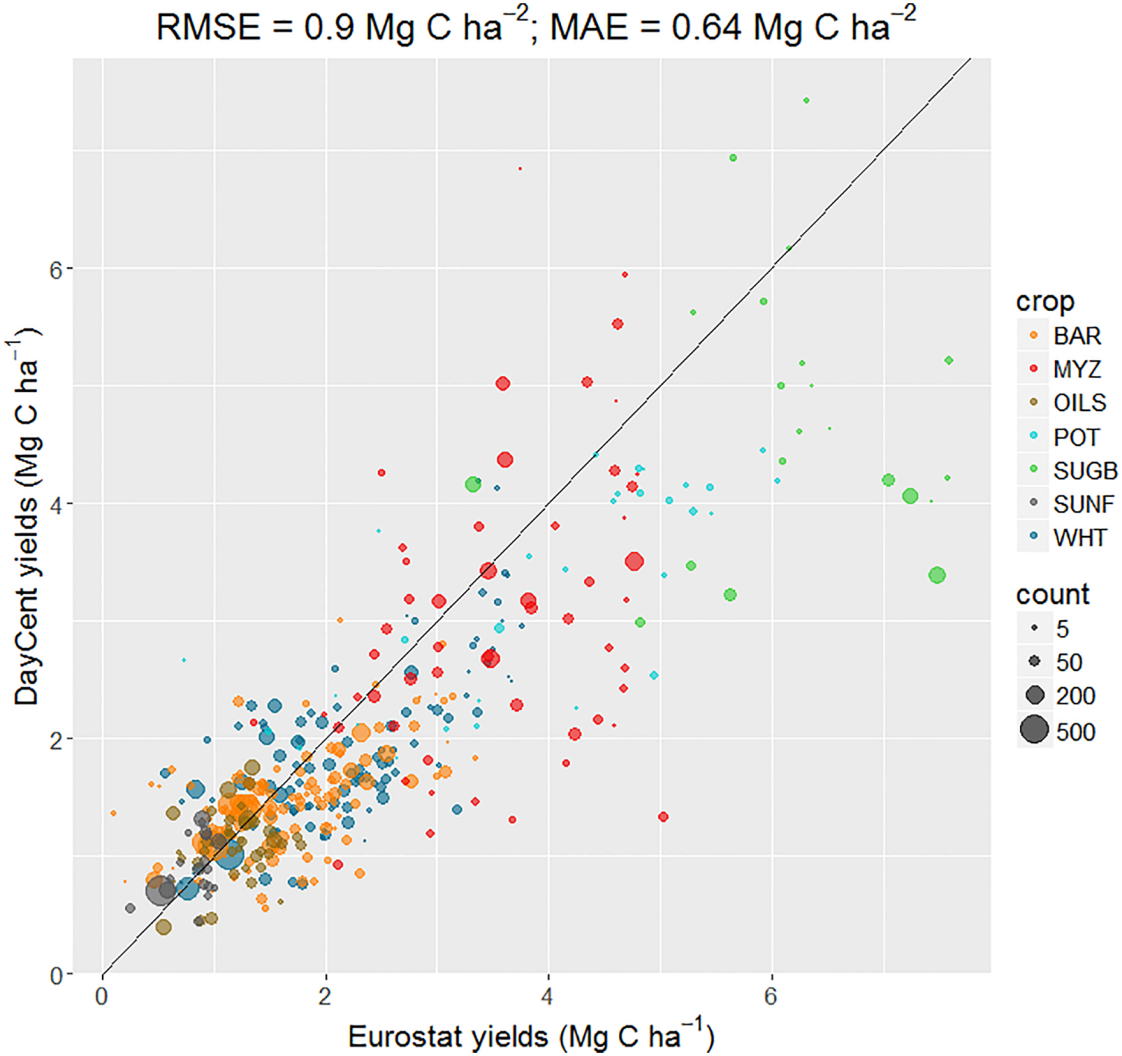
Simulated crop yields and comparison with Eurostat values, aggregated at NUTS2 level. The size of points represents the number of simulations for the following crops within each NUTS2 region. RMSE = root mean square error: MAE = mean absolute error.

The simulated yields of the main crops were generally in good agreement with published regional EU statistics, with an overall Root Mean Square Error (RMSE) of 0.9 Mg C ha^-1^ (Fig 2). Some NUTS2 regions were over-represented by LUCAS sites, especially for wheat, barley and oilseed crops. Other than for sugar beet, the deviation from the 1:1 line was noted in NUTS regions with a lower number of simulated values (and hence of LUCAS points), indicating a possible lack of statistical representativeness rather than a model bias. The Mean Absolute Error (MAE), which is less sensitive to outliers than RMSE, was 0.67 Mg C ha^-1^.

### Estimated soil N_2_O fluxes

The DayCent model simulates direct N_2_O fluxes from soils accounting for all emissions derived from the biogeochemical cycling of different N sources (inorganic and organic fertilizer, biological soil and crop fixation, N depositions, mineralization of soil organic carbon and plant litter).

**Fig 3:**
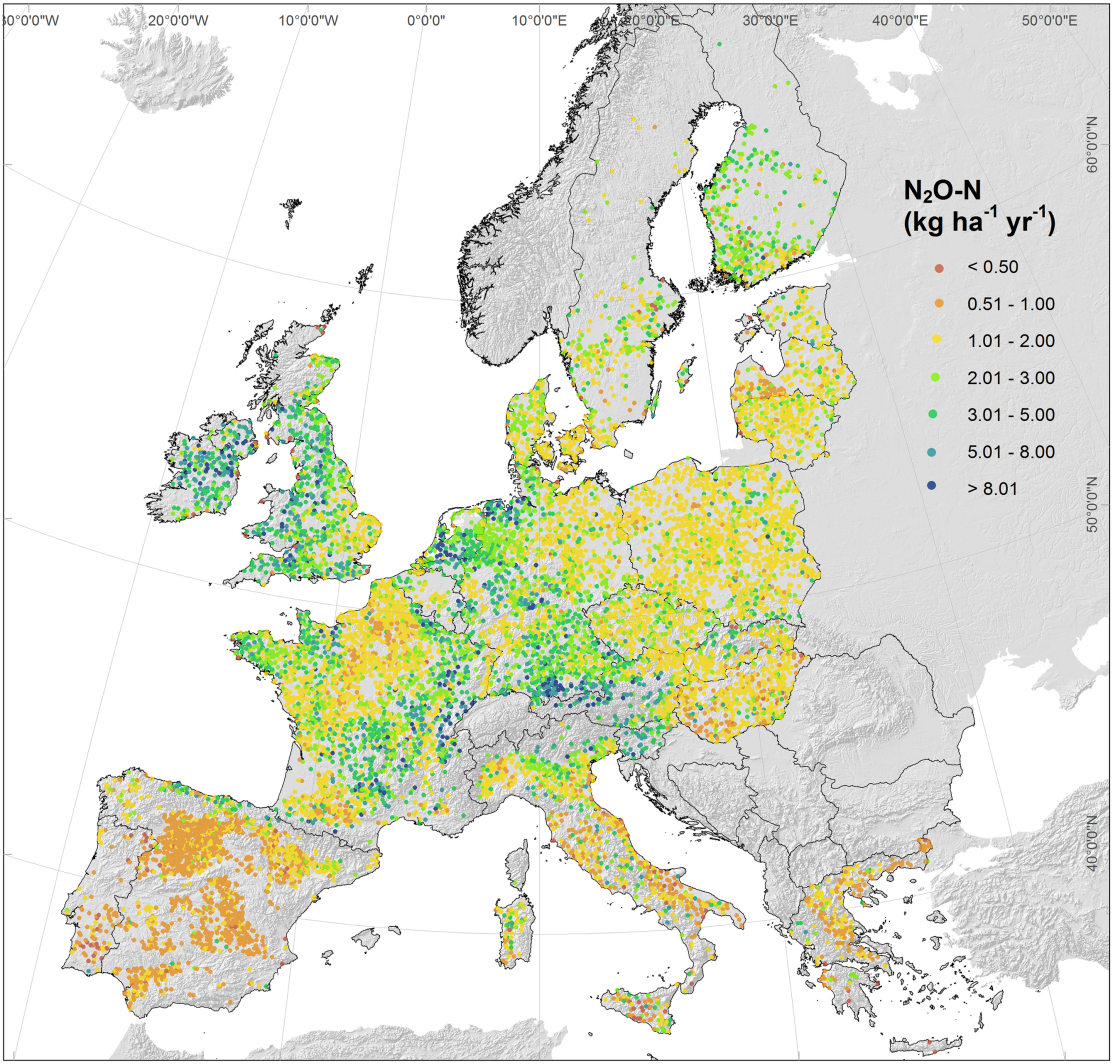
Simulated soil N_2_O-N emission rates (kg ha^-1^ yr^-1^) in LUCAS points.

Annual soil N_2_O fluxes followed a skewed distribution with mean and median values of 2.27 and 1.71 kg N ha^-1^ yr^-1^, respectively (1^st^ and 3^rd^ quartiles of 1.18, 2.63 kg N ha^-1^ yr^-1^). Geographically, lower emission rates were simulated in the Mediterranean regions where values below 1 kg N ha^-1^ yr^-1^ were frequent in Spain, Central-Southern Italy and Greece (Fig 3). Emission in eastern countries ranged mostly between 1 and 2 kg N ha^-1^ yr^-1^, while values above 3 kg N ha^-1^ yr^-1^ were often present in central Europe, UK and Ireland. Almost 8% of the LUCAS points displayed stimulated emissions higher than 5 kg N ha^-1^ yr^-1^.

**Fig 4:**
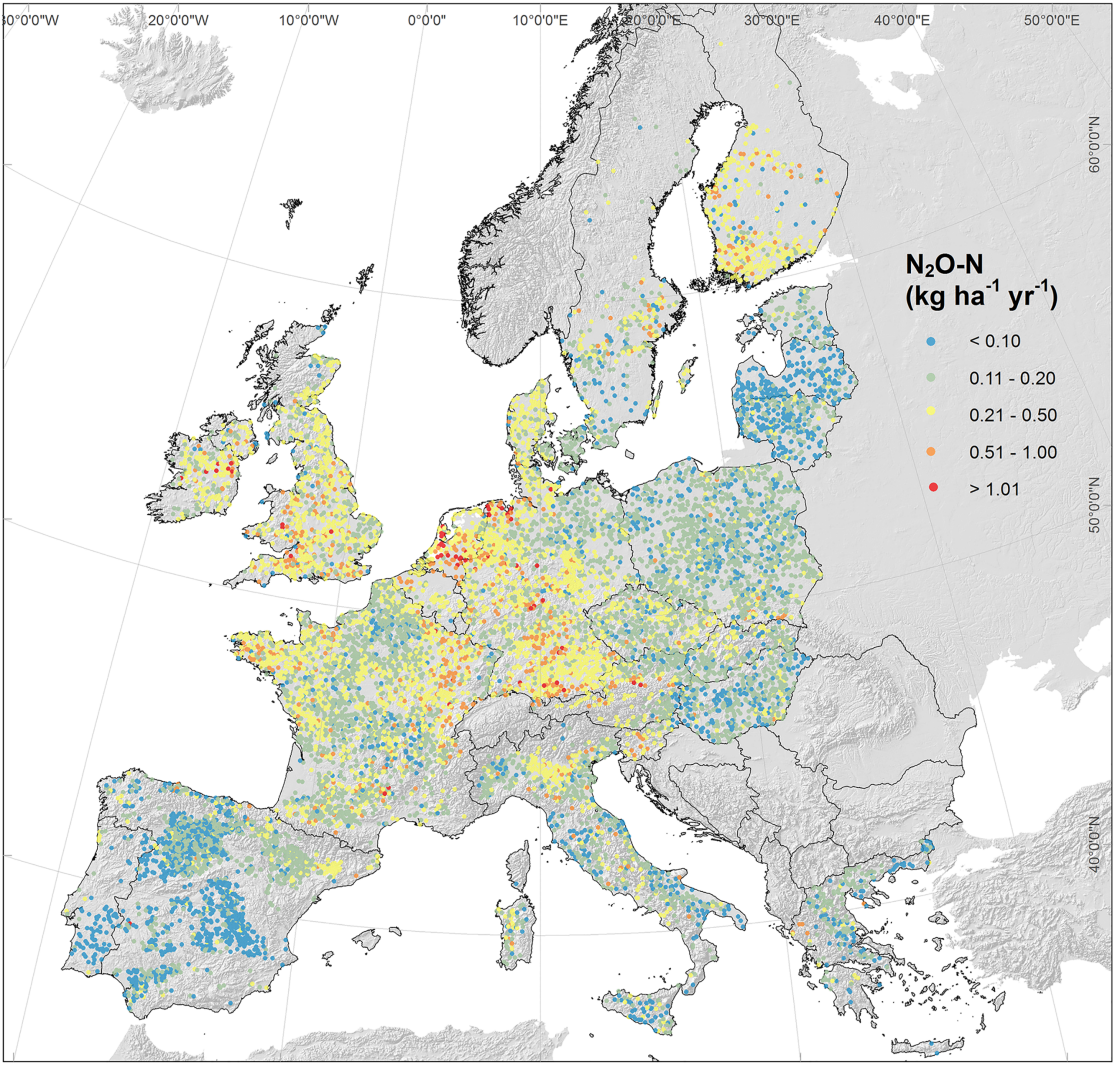
Model uncertainty of simulated soil N_2_O-N emission rates (kg ha^-1^ yr^-1^) in LUCAS points.

The uncertainty of the estimates was low in the Mediterranean and Eastern Europe, generally increasing with the emission rates elsewhere (Fig 4).

### Random Forest performance and upscaling

**Fig 5:**
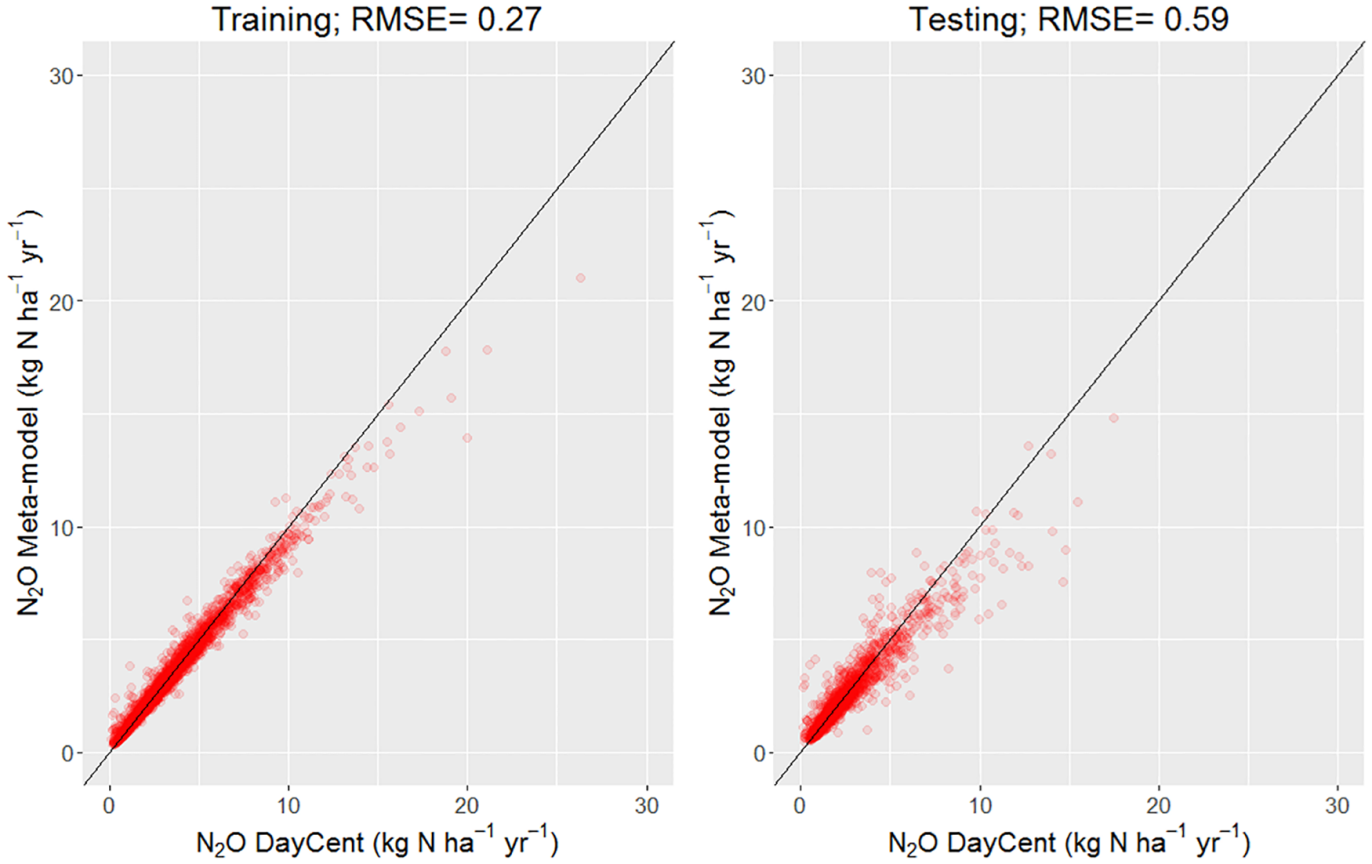
Random Forest meta-model evaluation (meta-model *vs* DayCent N_2_O rates) in the training (left) and testing dataset (right).

The Random Forest model explained a large part of the variance (89.3%) in the DayCent results, with lower RMSE in the training dataset and slightly higher in the validation dataset (0.27 and 0.59 kg N ha^-1^ yr^-1^, respectively) (Fig 5). The most important variable in the model, defined as the increase in mean of the squared prediction error when that variable is randomly permuted (%IncMSE), was the SOC stock (115.6%), followed by the clay content (90.0%), NPP (71.6%) and proportion of labile carbon (68.8%). Annual precipitation was ranked as the least important.

**Fig 6:**
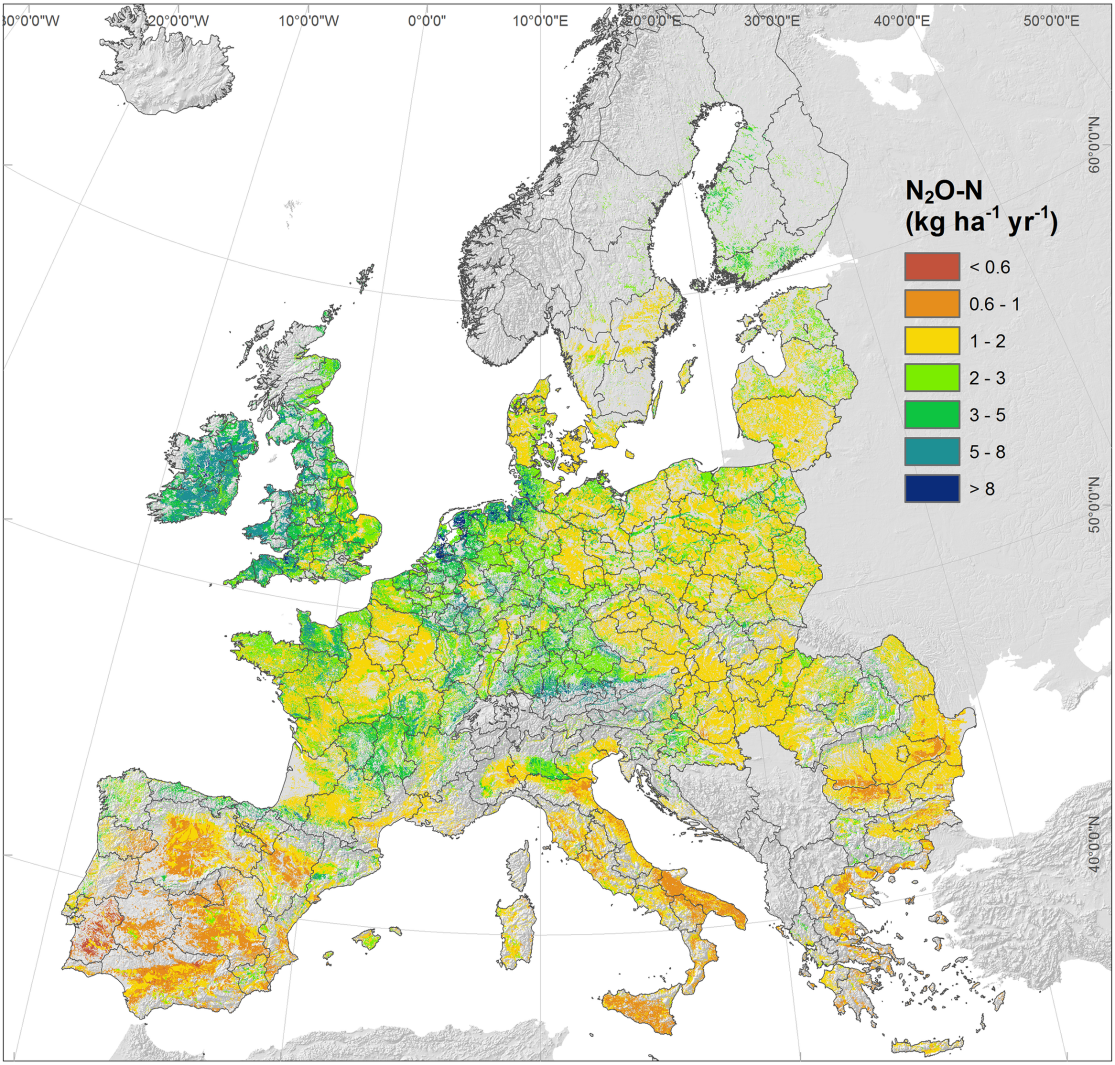
Map of soil N_2_O-N emissions based on Random Forest upscaling to a 1 km^2^ grid with the meta-model MT1. Administrative borders represent the EU basic regions (NUTS2) for the application of regional policies.

The application of the Random Forest model with the spatially continuous predictors gave spatial patterns and magnitudes of N_2_O emissions that were consistent with those resulting from simulations for LUCAS points (see Fig 6 for MT1). The cumulated emission in arable and grassland land use of EU28 countries simulated with MT1 amounted to 0.41 (0.076 2σ) Tg yr^-1^ of N_2_O-N, corresponding to 170.6 (±31.6) Tg of CO_2eq_ per year (using a Global Warming Potential of 265 at a time horizon of 100 years). The MT2 model resulted in a higher cumulated N_2_O emission equal to 195.0 Tg yr^-1^ of CO_2eq_, due to the higher SOC stock estimated by the ISRIC. Moreover, the direct upscaling of LUCAS-DayCent simulations by the agricultural land area coming from the Corine Land Cover gave a total emission of 157.7 Tg yr^-1^ of CO_2eq_, but it did not account for Croatia, Romania and Bulgaria excluded in 2009 LUCAS sampling.

**Fig 7:**
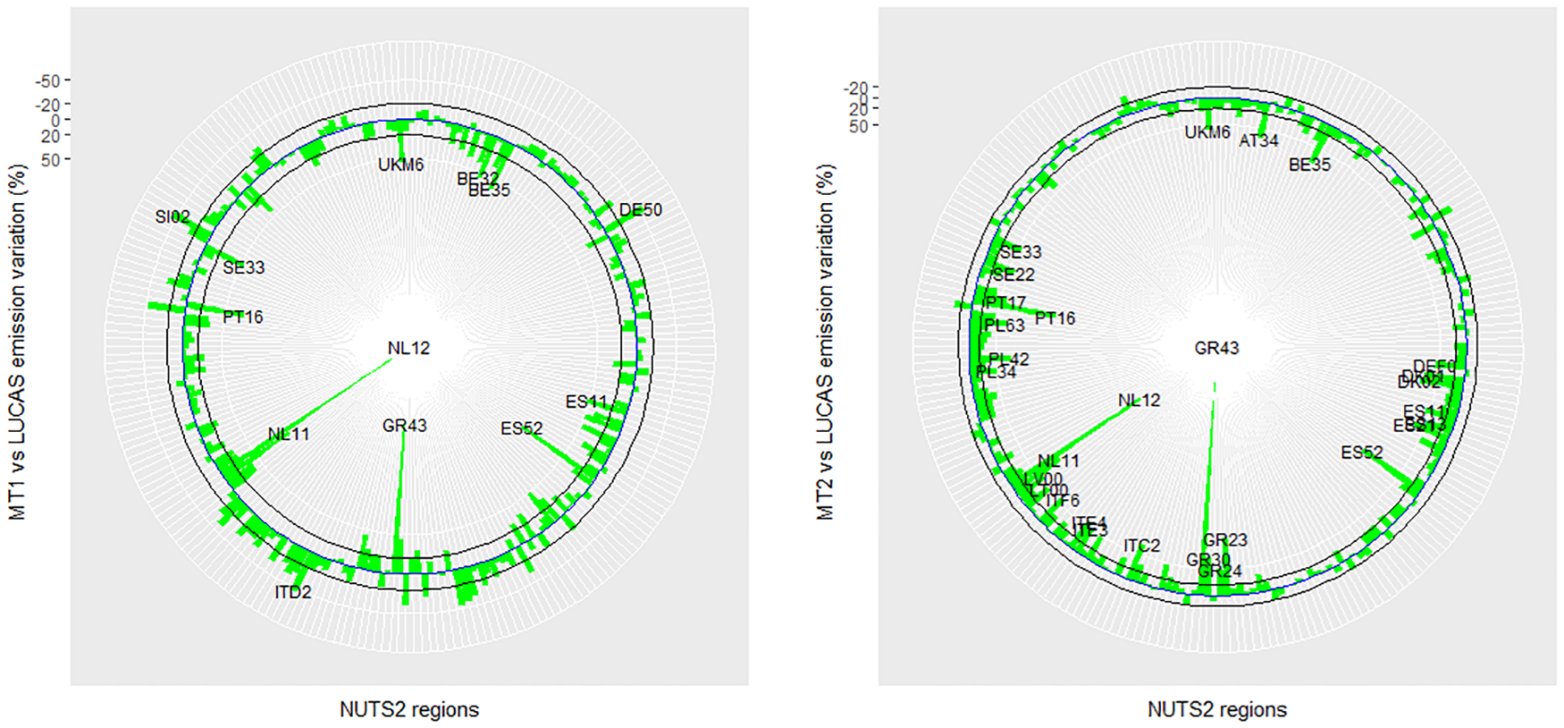
Percentage of variation in average N_2_O emissions between DayCent simulations at LUCAS sites and regional meta-model values for MT1 (upper) and MT2 (lower), at NUTS2 level. The blue line is the 0 value, while the black lines -20 and +20%. Labelled NUTS2 show variation >50% between average meta-model emissions compared to LUCAS points, with all green bars within the black lines meaning that the variation is within ±20%.

With the MT1 approach, 80 out of 234 NUTS2 resulted in emission variations (see Eq 1) outside a ±20% interval compared to the direct upscaling of LUCAS mean emissions at NUTS2. With the MT2 approach, that value was raised to 105 (Fig 7). Both meta-models highlighted some common regions (NL12, NL11, GR43, BE35, ES11, ES52, PT16, SE33, UKM6) where emissions varied by more than 50%.

## Discussion

Considering the objectives of the current CAP programme (2014–2020), the creation of a standardized land use and soil monitoring framework [3] is a fundamental step for monitoring the sustainability of agricultural systems and their environmental status as oriented by given policies and associated instruments. LUCAS soil information has been demonstrated to produce new high-resolution soil properties layers [34] or be useful to validate soil organic carbon inventories [27,35]. The latter are certainly a key component to monitoring policies that aim to sequester CO_2_, and thus contributing to meeting greenhouse gases emission reduction targets in agriculture, as defined in the proposal to integrate the land use sector into the EU2030 Climate and Energy Framework. However, the inclusion of N_2_O emissions assessment is a requisite for evaluating the full greenhouse gas balance of climate mitigation actions, due to the high climate relevance of N_2_O [36], but also in its role in depleting stratospheric ozone [37].

Stations with continuous N_2_O measurements or discrete air sampling sites can be used in top-down estimates of European N_2_O emissions [38]. Inverse models are continuously improving both in terms of spatial resolution and decreasing uncertainty and have shown that—at the national scale—uncertainties of agricultural N_2_O emissions estimated with the simple IPCC methods might be lower than generally assumed [38]. However, both top-down and IPCC methods will not be able to provide fine-grained estimates of N_2_O emission fluxes caused by environmental change, land use change, or changing management practices.

A better link between N_2_O emissions and changes in management and environmental conditions can be obtained by the use of biogeochemistry models, which are often run and/or calibrated against measured data at site level [39,40] and spatially upscaled by the use of meta-models [22,41]. However, the large variability in environmental conditions poses a challenge for process-based models if the number of experimental data used for their calibration is insufficient and do not cover the whole range of environmental conditions in the model application area [42]. Experimental field sites monitoring N_2_O fluxes from agricultural soils are usually financed by short-term projects of a few years and long-term time series are almost non-existent. While the number of available measurements is continuously increasing, for instance in Mediterranean regions [43], a thorough validation of regional estimates of N_2_O fluxes calculated with process-based model for agricultural soils in Europe is still missing.

In our study, the DayCent model predicted average N_2_O emissions of 2.27 (median 1.71) kg N ha^-1^ yr^-1^ in LUCAS points, corresponding to 2.2 kg N ha^-1^ yr^-1^ with the meta-model upscaling (MT1). These numbers are consistent with other model integrations such as INTEGRATOR [44] and MITERRA [45], that report N_2_O emissions of 1.1–2.4 (in arable land) and 2 kg N ha^-1^ yr^-1^ in the agricultural area of EU27 for the year 2000, respectively. Based on the above results and the outputs of other models (IDEAg, IMAGE, UNFCC), the most recent ‘European Nitrogen Assessment’ [46] reported total N_2_O emission rates for EU27 ranging between 0.33 and 0.43 Mt N yr^-1^, which are similar to our estimations of 0.41 and 0.47 Mt N yr^-1^ with MT1 and MT2, respectively. Moreover, total N_2_O emissions of 170.6 (±31.6) Tg of CO_2eq_ per year were higher than the direct N_2_O emissions from agricultural soils in the EU reported to the UNFCCC in 2015, averaging about 130 Tg of CO_2eq_. for the last decade [47] but based on lower tier accounting methodologies.

Certainly, our results incorporate different types of uncertainty associated with different methodological steps. Firstly, the process-based model uncertainty. Even though DayCent is one of the most used process-based models for estimating N_2_O fluxes [48,49], calibration of the model at experimental field sites in Europe is scarce and the model-bias is therefore largely unknown. The Random Forest approach was able to explain most of the variance with very low RMSE both in the training and testing dataset. In agreement with Perlman et al. [21] and Giltrap et al. [22], who developed a meta-model based on DNDC simulations, SOC content was the most explaining variable. This indicates a common model sensitivity to SOC of the most widely used biogeochemistry models, which seems partially supported by meta-analysis [50], as other factors such as pH or air temperature can have the same magnitude in response. In fact, the trade-off between increasing model complexity and additional parameters uncertainty followed by high computational time is likely the main constrain for fast modification of existing codes [51].

Secondly, the model input uncertainty. This uncertainty is high at LUCAS point level due to the lack of information on management practices. Although this aspect cannot be addressed as data are missing, the use of probability density functions can help to quantify the associated variance.

Input uncertainty is also added in the Random Forest upscaling process. When we changed the two most explaining variables (i.e. SOC and clay) in the spatial dataset, we obtained a 14% difference in total N_2_O emission at EU level, mainly due to the higher SOC stock values in MT2 compared to MT1 (S3 Fig).

At NUTS2 level, DayCent-LUCAS simulations and the two meta-models provided comparable information as variation was below 20% in the majority of the regions. The NUTS2 are administrative borders which represent the EU basic regions for the application of regional policies, therefore policy decisions are often evaluated on indicators aggregated at that territorial level. Under this perspective, simulations on LUCAS points may be representative for EU policy support, with possible exception in a few NUTS2 regions where both meta-models indicated a substantial bias. For instance, in the NL12 NUTS region, one of the areas with the highest variation, the six LUCAS sampling points were all close to the coastline; in addition, the average SOC stock on those locations was only one third of the estimates from both [30] and ISRIC datasets. This indirect verification may help to assess LUCAS representativeness that could be evidently improved by increasing the number of soil sample points or verifying potential sources of bias (point location/classification, analysis errors, etc.).

## Conclusion

There is substantial orientation in the EU to finance the LUCAS survey supporting various policy areas (agricultural, climate and environmental policies), in view of the multiple uses that such kind of data can provide (e.g. assess environmental changes, update European soil maps, validate soil models). We demonstrated how a model framework can be very useful to complement LUCAS information with N_2_O emissions. Some improvements are needed to retrieve more information about actual management practices that we tried to encompass by quantifying the associated uncertainty. Furthermore, a combination of intensive flux measurement sites to calibrate process-based models would be essential to increase model accuracy. This might enable the LUCAS survey to become not only a reference benchmark for monitoring environmental status and its change, but possibly also a supporting tool for reporting obligations, such as those under the UNFCCC. Definitely, a land use/soil survey framework feeding a state-of the-art and well calibrated biogeochemistry model has the potential to become a powerful tool to support environmental policies affecting land use and management in the EU.

## Supporting information

S1 Fig Average mineral and organic N fertilization applied to LUCAS points. (PDF)

S2 Fig Total (mineral + organic) average N fertilization applied to LUCAS points. (PDF)

S3 Fig Absolute difference in SOC stock (0–30 cm) sources, used as spatial predictors in meta-model MT1 and MT2. (PDF)
