# Supplementary material for: Complementing the topsoil information of the Land Use/Land Cover Area Frame Survey (LUCAS) with modelled N2O emissions
Source: PLoS One. 2017 Apr 27;12(4):e0176111. doi: 10.1371/journal.pone.0176111 (PMC5407635; doi:10.1371/journal.pone.0176111)
Supplement: S1 Fig — (PDF) [file pone.0176111.s001.pdf]

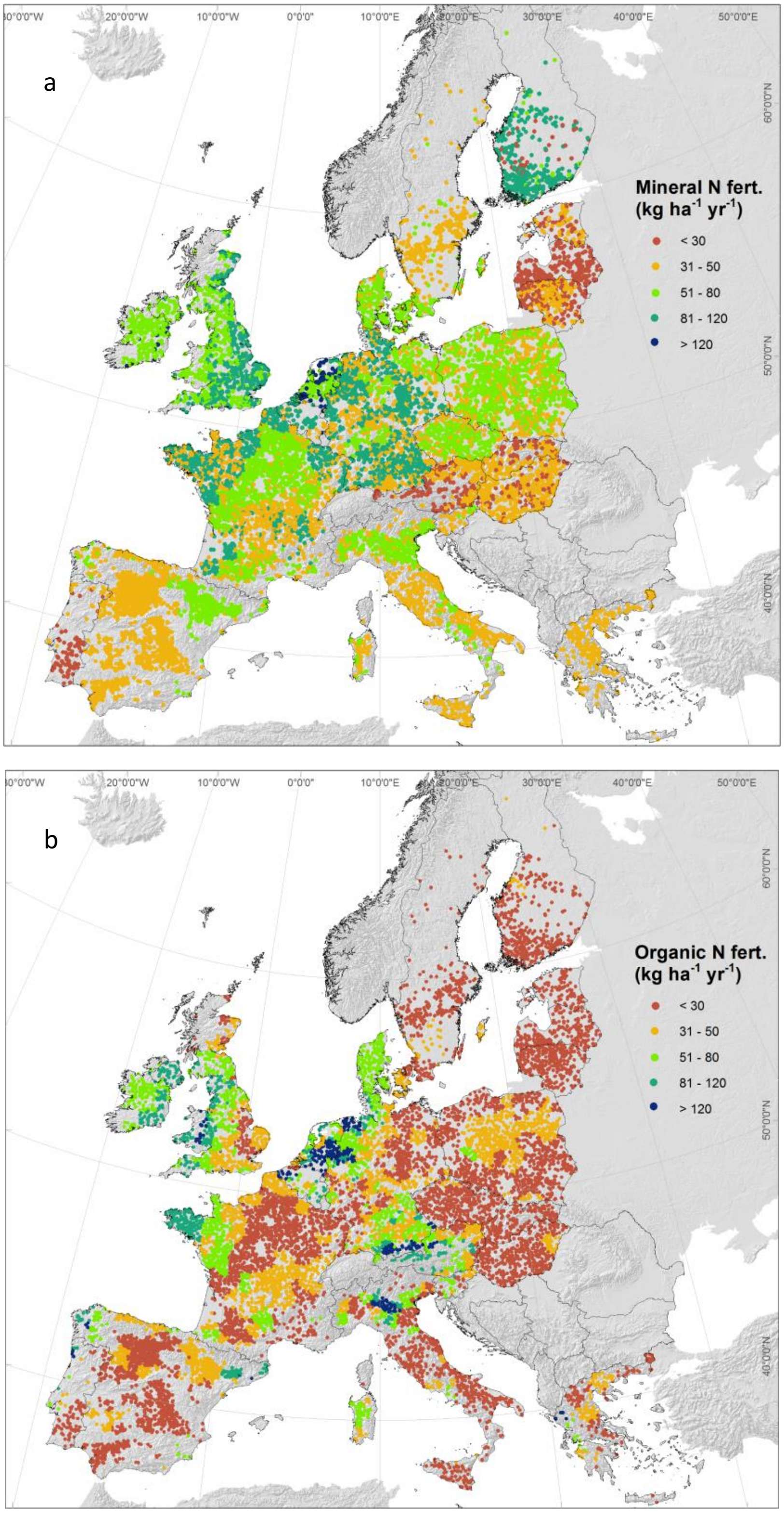

S1 Fig – Average (**a**) mineral and (**b**) organic N fertilization applied to LUCAS points. For the mineral fertilization, the values refer to the average N fertilization of the crop rotation since each crop (within the rotation) may have different rates based on agronomic requirement.
