# Supplementary figures and images for: Complementing the topsoil information of the Land Use/Land Cover Area Frame Survey (LUCAS) with modelled N2O emissions
Source: PLoS One. 2017 Apr 27;12(4):e0176111. doi: 10.1371/journal.pone.0176111 (PMC5407635; doi:10.1371/journal.pone.0176111)

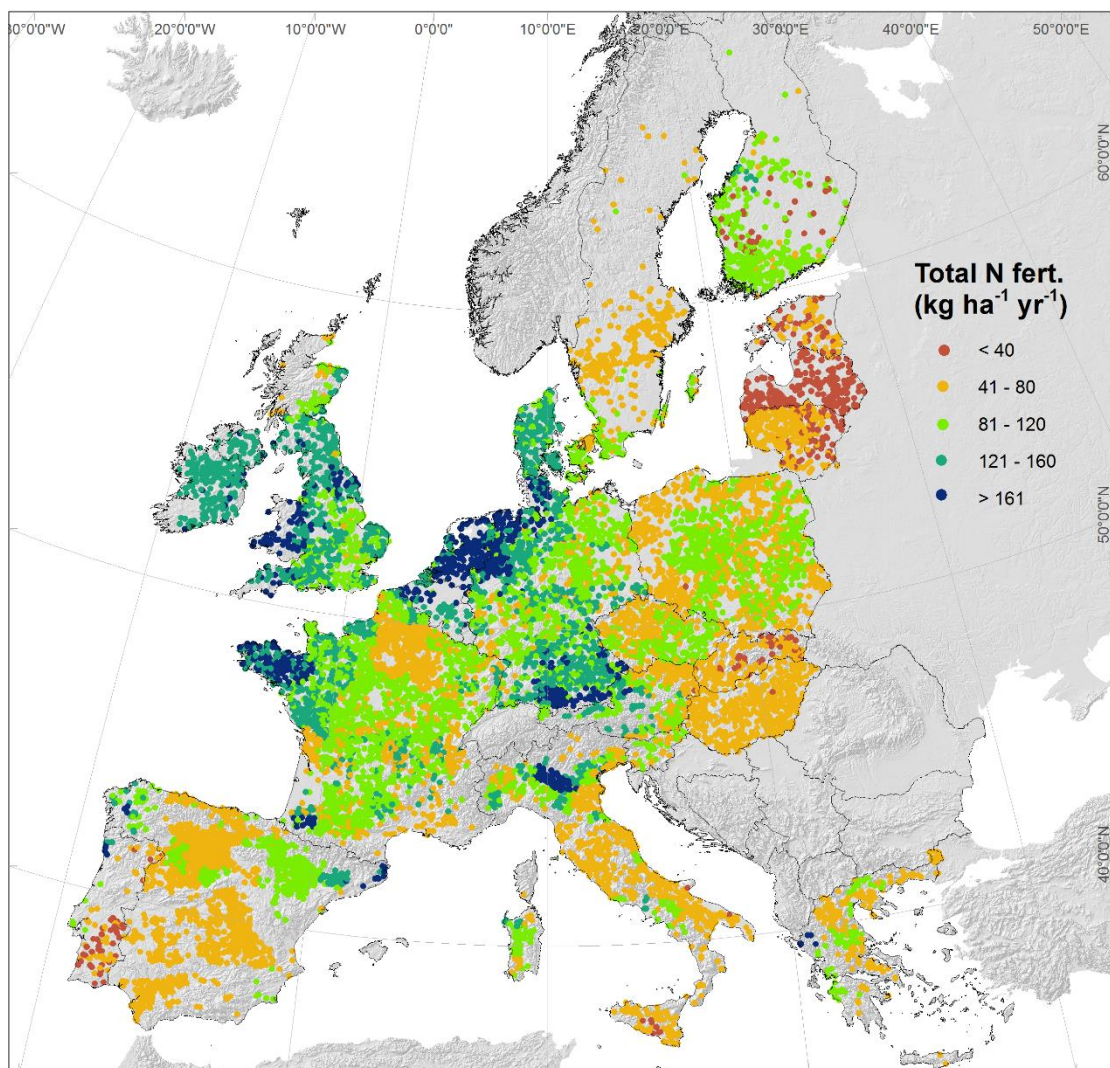

**S2 Fig** – Total (mineral + organic) average N fertilization applied to LUCAS points.

Supplement: S2 Fig — (PDF) [file pone.0176111.s002.pdf]
