# Supplementary material for: Complementing the topsoil information of the Land Use/Land Cover Area Frame Survey (LUCAS) with modelled N2O emissions
Source: PLoS One. 2017 Apr 27;12(4):e0176111. doi: 10.1371/journal.pone.0176111 (PMC5407635; doi:10.1371/journal.pone.0176111)
Supplement: S3 Fig — (PDF) [file pone.0176111.s003.pdf]

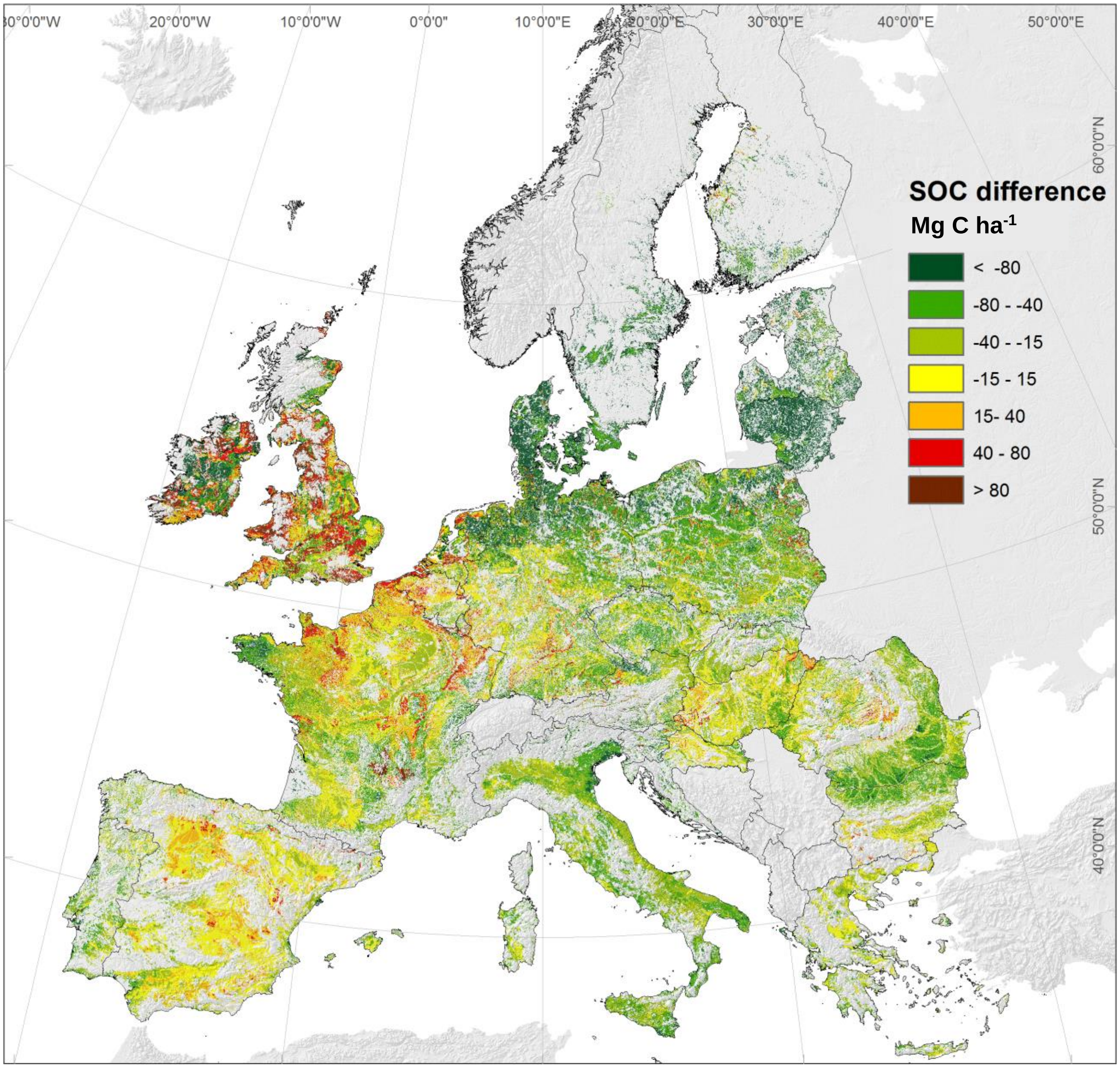

S3 Fig – Absolute difference in SOC stock (0-30 cm) between estimates from [1] and [2], used as spatial predictors in meta-model MT1 and MT2, respectively.

1. Lugato E, Paustian K, Panagos P, Jones A, Borrelli P. Quantifying the erosion effect on current carbon budget of European agricultural soils at high spatial resolution. Glob Chang Biol. 2016; 22(5): 1976–84.

2. Hengl T, de Jesus JM, MacMillan RA, Batjes NH, Heuvelink GBM, Ribeiro E, et al. SoilGrids1km — Global Soil Information Based on Automated Mapping. PLoS One. 2014; 9 (8):e105992.
